# Hand-assisted laparoscopic donor nephrectomy: 1864 cases in 15 years of experience

**DOI:** 10.55730/1300-0144.5438

**Published:** 2022-06-18

**Authors:** Bilal GÜNAYDIN, Taha UÇAR, Emre ARPALI, Başak AKYOLLU, Serkan AKINCI, Cihan KARATAŞ, Kenan ÖZTORUN, Burak KOÇAK

**Affiliations:** 1 Department of Urology, Niğde Ömer Halis Demir University School of Medicine, Niğde, Turkey; 2 Department of Urology, Niğde Ömer Halis Demir University Research and Training Hospital, Niğde, Turkey; 3 Koç University Hospital Organ Transplant Center, İstanbul, Turkey; 4 Department of Urology, Memorial Hizmet Hospital, İstanbul, Turkey; 5 Department of Urology, Koç University School of Medicine, İstanbul, Turkey

**Keywords:** Hand-assisted, laparoscopic, donor, nephrectomy, complication

## Abstract

**Background/aim:**

To evaluate hand-assisted laparoscopic donor nephrectomy (HALDN) in terms of intraoperative and postoperative results.

**Materials and methods:**

After institutional review board approval was obtained, a total of 1864 HALDN operations performed between March 2007 and January 2022 were retrospectively analyzed. Age, sex, body mass index (BMI), status of smoking and presence of previous abdominal surgery, laterality, operative time, transfusion requirement, port counts, length of extraction incision, time until mobilization, time until oral intake, donor serum creatinine levels before and one week after the surgery, length of postoperative hospital stay, intraoperative complications, and postoperative recovery and complications were recorded and statistically analyzed. Multiple renal arteries, BMI, right nephrectomy and male sex were also separately evaluated as risk factors for complications and operative time.

**Results:**

A total of 825 (44.26%) male and 1039 (55.74%) female patients were enrolled in the study. The mean age of the patients was 45.79 ± 12.88 years. There were a total of 143 complications (7.67% of the total 1864 cases) consisting of 68 (3.65%) intraoperative and 75 (4.02%) postoperative complications. Open conversion was necessary for 10 patients (0.53%) to manage intraoperative complications. Reoperation was needed for 1 patient due to bleeding 6 h after the operation. Multiple renal arteries were a risk factor for intraoperative complications and prolonged operative time. Right nephrectomy and male sex were also related with longer operative times.

**Conclusions:**

HALDN is a safe procedure associated with low complication rates.

## 1. Introduction

Kidney transplantation is the best treatment option for end-stage renal disease. As minimally invasive surgery has become more popular, living donor nephrectomy has evolved from open surgery to laparoscopic and robotic surgery. Laparoscopic donor nephrectomy (LDN) has been accepted as the standard method of kidney procurement from live donors [1].

Minimally invasive surgical techniques have the advantages of less postoperative pain, shorter hospital stay, less incisional morbidity, shorter recovery time and better cosmetic results [2]. Since donor nephrectomy patients are healthy volunteers who assume surgical risks not for their own benefits, utilizing the most appropriate surgical technique with maximum perioperative and postoperative comfort may help decrease the concerns of potential donor candidates.

LDN was first described by Ratner and colleagues [3]. Modifications of the standard approach have also been developed to help facilitate this complex laparoscopic procedure after the inception of the standard technique. Hand-assisted laparoscopic donor nephrectomy (HALDN), first adapted in 1998 by Wolf et al., has been chosen by many surgeons for the proposed advantages including shorter operative time, a shorter learning curve related to the presence of robust tactile feedback, the ability to manually assist in dissection, and ease of obtaining hemostasis by manual compression of bleeding vessels to prevent a possible open conversion [4].

This study presents our 15 years of experience with HALDN in 1864 patients, underlying the technical experience of our center, as well as reviewing the incidence and type of perioperative and postoperative complications encountered.

## 2. Materials and methods

### 2.1. Patient selection & data collection

This study included 1864 consecutive kidney donors who underwent HALDN procedures between March 2007 and January 2022. All medical data were collected retrospectively from a single transplant group. The following parameters were recorded: donor age, sex, body mass index (BMI), status of smoking and presence of previous abdominal surgery, laterality, operative time, transfusion requirement, port counts, length of extraction incision, time until mobilization, time until oral intake, donor serum creatinine levels before and one week after the surgery; 1^st^ hour and 24^th^ hour VAS score, length of postoperative hospital stay, intraoperative complications, and postoperative recovery and complications.

Multiple renal arteries, BMI ≥ 30, right LDN and male sex were picked as risk factors for complications and compelling factors for the procedures according to the current literature [5]. These three variables and male sex were investigated in terms of intraoperative complication rates and operative time.

### 2.2. Operative technique

The standard approach was HALDN, and all donor nephrectomies were performed by the same surgical team with the same standard technique [6]. We usually placed the hand port through a 6 or 7 cm lower quadrant incision. One 5 mm camera port was placed at a point on the midclavicular line close to the costal arch and another 12 mm instrumental port was inserted lateral to the rectus sheath and 10 cm cranial to the 5 mm port. All the surgical procedures were performed by a dedicated team of 3 experienced surgeons. The master surgeon (BK) trained 2 other surgeons (CK, EA) and supervised them throughout most of the procedures.

### 2.3. Grading system for complications of LDN

Kocak’s modified Clavien classification system for HALDN procedure was used to grade all intraoperative and postoperative complications as a standard method [7]. The nonlife threatening complications were classified as grade 1, complications with no residual disabilities were classified as grade 2, complications occurring residual disabilities such as organ resection were classified as group 3 and the complications causing death or renal failure were grouped as grade 4.

### 2.4. Statistical analysis

SPSS version 25 was used for statistical analysis. All numerical data were extracted as the mean ± standard deviation. Chi-square test was used to compare complications, open conversions and nonnumerical demographic data. Mann-Whitney U test was used to compare numerical demographic data, port counts, length of incision, operative time, time until mobilization, and time until oral intake after completing homogeneity evaluation. The odds ratio for multiple renal arteries, BMI, right LDN, and male sex in terms of complication rates were also calculated with Fisher’s exact probability test.

## 3. Results

### 3.1. Demographic data

There were a total of 825 (44.26%) male, 1039 (55.74%) female patients enrolled. The mean age of the patients was 45.79 ± 12.88 years (range: 18–84 years). The mean BMI of the patients was 27.58 ± 4.5 kg/m^2^. A total of 163 (8.7%) right and 1701 (91.2%) left HALDN were performed. All demographic data are summarized in Table 1.

### 3.2. Intraoperative and postoperative data

The mean operative time was 86.68 ± 36.31 min (median: 80; range: 25–270 min). The mean length of hospital stay was 2.00 ± 1.15 days. The mean time to oral intake was 7.28 ± 1.1 (median: 7; range: 3–25 h) h. A total of 398 (21.3%) patients were with multiple renal arteries. The mean preoperative and postoperative day 7 serum creatinine values of the donors were 0.74 ± 0.15 mg/dL and 1.15 ± 0.27 mg/dL, respectively. Postoperative mean first hour VAS score was 4.46 ± 2.30 and postoperative 24-h VAS score was 0.49 ± 0.77. The mean port count was 2.19 ± 0.58 (median: 2; range: 1–5), the mean length of incision was 6.92 ± 0.83 cm (median: 7; range: 5–25 cm).

### 3.3. Intraoperative complications

All intraoperative complications are summarized in Table 2. There were a total of 68 (3.65%) intraoperative complications in our study. The most common intraoperative complications were renovascular injuries. All renal artery, renal vein, lower or upper renal pole artery and vein and stapler incidents were grouped as renovascular complications. There were 27 renovascular complications (40.2% of total intraoperative complications), including 3 stapler incidents. There were 6 vascular complications (8.90% of total intraoperative complications), including 2 vena cava and 4 lumbar vein injuries. Renovascular complications were mostly managed laparoscopically (74% vs. 26%). Vascular complications were also managed laparoscopically. One of six (16.60%) vascular complications -inferior vena cava injury- was managed with open conversion.

There were 10 open conversions (0.53%). Eight of these conversions were related to renovascular and vascular complications: 2 renal artery injuries, 1 renal vein injury, 1 inferior vena cava injury, 1 lower pole artery injury, and 3 stapler incidents. The other 2 conversions were due to adrenal bleeding. The estimated blood loss for most of the kidney donors in this study was negligible; only 4 (0.21%) of patients required blood transfusion.

### 3.4. Postoperative complications

In total, there were 75 (4%) postoperative complications (angina pectoris, 1; atrial fibrillation, 5; elevated liver enzymes, 8; elevated bilirubin, 1; toxic hepatitis, 1; chylous ascites, 3; prolonged ileus, 12; incisional hernia, 5; seroma, 3; infection at port side, 1; subcutaneous hematoma, 3; selulitis, 1; bleeding, 1; rhabdomyolysis, 1; hemoglobin loss less than 2 g/dL, 1; gluteal hematoma, 1; acute renal failure, 1; urinary retention, 4; urinary tract infection, 4; left orchialgia, 13; right orchialgia, 1; inguinal orchiectomy, 1; scrotal hematoma, 1; hematuria, 1; acute prostatitis, 1. The only complication requiring reoperation was bleeding from the lumbar vein that occurred 6 h after HALDN. Postoperative complications are summarized in Table 3.

### 3.5. Grading of complications

We observed 118 (6.33%) complications using the proposed classification system; 41 (34.7%) of the complications were defined as grade 1, 19 (16.10%) as grade 2a, 40 (33.80%) as grade 2b, 10 (8.40%) as grade 2c, and 8 (6.70%) classified as grade 3. There were no grade 4 complications (Table 4).

### 3.6. Donors with multiple renal arteries

There were a total of 372 patients with multiple renal arteries. The intraoperative complication rate was 5.10% (20 patients) for patients with multiple renal arteries versus 1.70% (25 patients) for patients with single renal arteries (p < 0.01). Multiple renal arteries were defined as a risk factor for intraoperative complications with a 2.99 odds ratio. Multiple renal arteries were also related with longer operative time compared to single renal arteries.

### 3.7. Obese donors

A total of 589 (31.5%) donors had a BMI greater than 30 kg/m^2^. Obesity was neither a risk factor for intraoperative complications nor a factor for prolonged operative time.

### 3.8. Right LDN

In this series of HALDN patients, right donor nephrectomy was performed with a rate of 8.70% (163 patients). Mean operative time was longer for right donor nephrectomies when compared to left donor nephrectomies with durations of 106.32 ± 30.27 min vs. 84.71 ± 36.22 min, respectively (p < 0.01). Right donor nephrectomy was not a risk factor for intraoperative complications (odds ratio: 1.90).

### 3.9. Sex

Mean operative time was longer for male donors compared to female donors with durations of 90.52 ± 34.95 min vs. 83.67 ± 37.08 min, respectively (p < 0.01). Male sex was not a risk factor for intraoperative complication rate.

### 3.10. Parameters affecting intraoperative complication rates and operative time

Multiple renal arteries were defined as a risk factor with a 2.99 odds ratio. BMI ≥ 30, right nephrectomy, and male sex was not a risk factor for complications (Table 5). Multiple renal arteries, right nephrectomy, and male sex were related to longer operative times. BMI was not related to prolonged operative time (Table 6).

## 4. Discussion

Perioperative and postoperative outcomes of 1864 consecutive HALDN cases from a single group are summarized in this study. The overall complication rates of our series were 7.6% consisting of 68 (3.65%) intraoperative and 75 (4.02%) postoperative complications.

Strivastava et al. reported 8.60% complication rates in 1430 cases of LDN series [8]. According to the literature, the complication rates of LDN vary between 5.46%–8.60% [6–9]. In a meta analysis conducted in 2018, which consists of 21 HALDN studies including a total of 1393 patients, the total complication rate was reported to be 9.40% [10]. In the same metaanalysis intraoperative complication rate was 3.10%, the postoperative complication rate was 6.90% and the open conversion rate was 1.83%. Our total complication rate was lower than the literature at 6.30% and the intraoperative complication rate was 3.50% .

The vast majority of the intraoperative complications were vascular and renovascular complications. There were 10 open conversions (0.53%). Eight of these conversions were related to renovascular and vascular complications. The other 2 conversions were due to nonvascular complications. Our open conversion rate was remarkably low when compared to the literature. An explanation for this low rate could be the management of intraoperative complications, particularly vascular and renovascular ones, laparoscopically avoiding an open conversion.

We also evaluated the operative time as an indicator that determines the difficulty of an operation. Multiple renal arteries, right LDN, and male sex were prolonging the operative time while BMI had no effect on operative time. The variables that prolonged operative time were similar to the literature besides BMI, which was not a compelling factor for our patients contrary to the literature. According to the literature, obesity was related with longer operative time and longer length of hospital stay. The complication rates and morbidity were not related to obesity [11,12]. However, in our study, the variables prolonging the operative time were not related with the intraoperative complication rates except for multiple renal arteries. The presence of multiple renal arteries was found to be a significant risk for intraoperative renovascular complications in our study. Multiple renal arteries were also accused in terms of increased risk of vascular complications by many authors. According to the current opinion, LDN can be applied safely with or without multiple renal arteries owing to surgical advancements and accumulation of experience. However, we would like to draw attention to multiple renal arteries in terms of increased risk of intraoperative complication rates in a recent study also conducted by our group [13].

Right LDN is related with increased operative time, hospital stay, and increased allograft failure in the literature [14–16]. The vascular features of the right kidney are the main complicating factor with short and thin walled renal vein and retrocaval renal artery [14]. In another retrospective study comparing right and left donor nephrectomy in terms of complication rates and operative time which included 527 patients consisting of 423 (80.20%) left and 104 (19.70%) right donor nephrectomy, right donor nephrectomy was related with increased operative time and complication rates [16]. According to a randomized controlled trial, recently developed laparoscopic techniques lead to shorter operative time even shorter than left donor nephrectomy [15]. But most of the studies that had lower operative time and less complication rates for right donor nephrectomy had a small sample size with limited follow up time. According to our experience right donor nephrectomy had longer operative times with comparable complication rates with left LDN.

Since we entirely focused on donor morbidity in our present study and decided not to report recipient outcomes, long-term graft survival and recipient data were not presented here. We believe that the retrospective nature of our study is the major limitation. To our knowledge, this is one of the largest published HALDN series in the world including nearly 2000 patients.

HALDN is a proven safe procedure for donor nephrectomy with lots of reliable evidence in the literature. However, the contribution of larger series to current literature is undeniable. Multiple renal arteries were found to be a risk factor for intraoperative complications and male sex, multiple renal arteries, and right donor nephrectomy were related with longer operative times.

## Figures and Tables

**Table 1:** Demographics.

Variables	
**Age** (years ± SD ) (Range)	45.79 ± 12.88 (18.00–84.00)
**Sex**	
Male (years/percentages)	825 (44.26%)
Female (years/percentages)	1039 (55.74%)
**BMI** (kg/m^2^) ± SD (Range)	27.58 ± 4.59 (14.87–41.70)
**Smoking status**	
Smoker (years/percentages)	889 (47.69%)
Nonsmoker (years/percentages)	975 (52.31%)
**History of abdominal surgery**	
Present (numbers/percentages)	668 (35.84%)
Absent (numbers/percentages)	1196 (64.16%)
**Laterality**	
R (numbers/percentages)	163 (8.74%)
L (numbers/percentages)	1701 (91.26%)

**Table 2:** Intraoperative complications.

Complication	Number of patients	Management
Renovascular complications	20	Laparoscopic management
Renovascular complications	4	Conversion
Stapler incident	3	Conversion
Inferior vena cava injury	1	Conversion
Vena cava Injury	1	Laparoscopic management
Lumbar vein injury	4	Laparoscopic management
Meso injury	8	Laparoscopic management
Adrenal bleeding	2	Conversion
Adrenal bleeding	14	Laparoscopic management
Serosal bowel injury	4	Laparoscopic management
Splenic capsule tear	1	Laparoscopic management
Subcapsular hematoma	6	Expectant

**Table 3:** Postoperative complications.

Complication	Number of patients
Angina pectoris	1
Atrial fibrillation	5
Elevated liver enzymes	8
Elevated bilirubin	1
Toxic hepatitis	1
Chylous ascites	3
Prolonged ileus	12
Incisional hernia	5
Seroma	3
Infection at port side	1
Subcutaneous hematoma	3
Selulitis	1
Bleeding	1
Rhabdomyolysis	1
Hemoglobin loss less than 2 G/DL	1
Gluteal hematoma	1
Acute renal failure	1
Urinary retention	4
Urinary tract infection	4
Left orchialgia	13
Right orchialgia	1
Inguinal orchiectomy	1
Scrotal hematoma	1
Hematuria	1
Acute prostatitis	1

**Table 4:** Complications graded by severity.

Complications	% of all complications (n = 118)	% of total series (n = 1864)
Grade 1	34.75% (n = 41)	2.20%
Grade 2	57.63% (n = 68)	3.65%
2a	16.10% (n = 19)	1.02%
2b	33.90% (n = 40)	2.15%
2c	8.47% (n = 10)	0.54%
Grade 3	6.78% (n = 8)	0.43%
Grade 4	0	0

**Table 5:** Risk estimate for intraoperative complications.

Variable	Odds ratio*	95% confidence interval (Lower-Upper)
Multiple renal arteries*	2.99	1.65–5.41
BMI ≥ 30	0.79	0.44–1.42
R LDN	1.90	0.86–4.40
Male sex	0.82	0.45–1.44

* p-value: < 0.01;

Risk Analysis-Odds Ratio

**Table 6:** The factors affecting operative time.

Variable	Median (Min-Max) (min)	p-value *	95% confidence interval (Lower- Upper)
Multiple renal arteries	100 (50–190) vs. 80 (25–225)	<0.01	−12.9–−4.7
BMI ≥ 30	80 (25–270) vs. 80 (25–225)	0.65	2.6–4.5
R LDN	100 (50–190) vs. 80 (25–270)	<0.01	16.61–26.68
Male sex	90 (25–238) vs. 80 (25–270)	<0.01	3.45–10.17

* Mann Whitney U Test
